# Generation of a zebrafish neurofibromatosis model via inducible knockout of *nf2a/b*

**DOI:** 10.1242/dmm.050862

**Published:** 2024-12-04

**Authors:** Ayyappa Raja Desingu Rajan, Yuanyun Huang, Jan Stundl, Katelyn Chu, Anushka Irodi, Zihan Yang, Brian E. Applegate, Marianne E. Bronner

**Affiliations:** ^1^ Division of Biology and Biological Engineering, California Institute of Technology, Pasadena, CA 91125, USA; ^2^ Faculty of Fisheries and Protection of Waters, University of South Bohemia in Ceske Budejovice, 38925 Vodnany, Czech Republic; ^3^ University of Cambridge School of Clinical Medicine, Addenbrooke's Hospital NHS Foundation Trust, Cambridge CB2 0QQ, UK; ^4^ University of Southern California, Caruso Department of Otolaryngology-Head and Neck Surgery, Los Angeles, CA 90033, USA; ^5^ University of Southern California, Alfred Mann Department of Biomedical Engineering, Los Angeles, CA 90089, USA

**Keywords:** Schwann cells, Cancer, Inducible knockout, Meninges, Neurofibromatosis type 2, Zebrafish

## Abstract

Neurofibromatosis type 2 (NF-2) is a dominantly inherited genetic disorder that results from variants in the tumor suppressor gene, neurofibromin 2 (*NF2*). Here, we report the generation of a conditional zebrafish model of neurofibromatosis established by inducible genetic knockout of *nf2a/b*, the zebrafish homologs of human *NF2*. Analysis of *nf2a* and *nf2b* expression revealed ubiquitous expression of *nf2b* in the early embryo, with overlapping expression in the neural crest and its derivatives and in the cranial mesenchyme. In contrast, *nf2a* displayed lower expression levels. Induction of *nf2a/b* knockout at early stages increased the proliferation of larval Schwann cells and meningeal fibroblasts. Subsequently, in adult zebrafish, *nf2a/b* knockout triggered the development of a spectrum of tumors, including vestibular Schwannomas, spinal Schwannomas, meningiomas and retinal hamartomas, mirroring the tumor manifestations observed in patients with NF-2. Collectively, these findings highlight the generation of a novel zebrafish model that mimics the complexities of the human NF-2 disorder. Consequently, this model holds significant potential for facilitating therapeutic screening and elucidating key driver genes implicated in NF-2 onset.

## Teaser

**Editor's choice:** Zebrafish *nf2a/b* genes are expressed in tissues involved in neurofibromatosis type II (NF-2). Inducible knockout of *nf2a/b* triggers the development of a spectrum of tumors, mirroring the manifestations observed in patients with NF-2.

## INTRODUCTION

Neurofibromatosis type 2 (NF-2) is an autosomal-dominant disorder resulting from germline/mosaic variants in the neurofibromin 2 (*NF2*) tumor suppressor gene, leading to multiple benign tumors in the nervous system and along peripheral nerves. Despite its benign nature, NF-2-associated tumors can lead to neurological deficits such as early-onset hearing loss, issues with balance, cataracts, seizures, pain and problems with facial expressions. NF-2 tumors are primarily composed of Schwannomas, meningiomas, ependymomas, astrocytomas and, infrequently, neurofibromas, retinal hamartomas and intraorbital tumors (Campian and Gutmann, 2017; Coy et al., 2020; Ren et al., 2021; Tamura, 2021). Although the estimated incidence of germline variants in NF-2 is ∼1 in 33,000, NF-2 also has one of the highest rates of mosaicism, with reports suggesting that half of all individuals with NF-2 variants have *de novo* genetic alterations (Chen et al., 2022). To date, the best treatment options for NF-2 are surgical removal, chemotherapy and radiation therapy.

The *NF2* gene is a member of the ERM (ezrin, radixin, moesin) family of cell adhesion protein-encoding genes and codes for the protein merlin, which acts as a tumor suppressor. Merlin functions as a membrane–cytoskeleton linker that inhibits cellular proliferation via contact-dependent regulation of various signaling pathways, including WNT/β-catenin, Notch, RAS, RAC/RHO, TGF-β, Hippo and receptor tyrosine kinases (Hamaratoglu et al., 2006; Okada et al., 2007; Zhang et al., 2010). During mouse embryonic development, *Nf2* is highly expressed in various tissues. In the adult, its expression is predominantly observed in Schwann cells, meningeal cells, neurons, oligodendrocytes, mesothelium, optic neuroepithelial compartments and lens fiber cells (den Bakker et al., 1999; Moon et al., 2018; Toledo et al., 2018).

Modeling the plethora of phenotypes seen in NF-2 patients has been challenging, as biallelic knockouts of *Nf2* in mice are lethal owing to failure to initiate gastrulation (McClatchey et al., 1997). Heterozygous/hemizygous knockouts of *Nf2* are cancer prone and demonstrate a tumor spectrum that differs significantly from that observed in NF-2 patients; they do not develop Schwannomas, a prominent feature of NF-2 (Giovannini et al., 2000; McClatchey et al., 1998). To avoid these issues, conditional knockouts for *Nf2* have been generated in specific cell lineages. Conditional biallelic knockout of *Nf2* using P0-Cre transgenic mice leads to the development of Schwannomas, cataracts, and tumors in tissues with neural crest-derived components. However, these mice do not develop vestibular Schwannomas, the hallmark tumors of human NF-2 (Giovannini et al., 2000). Mice with conditional inactivation of the *Nf2* gene in leptomeningeal cells via subdural injection of *adCre* in *Nf2^flox2/flox2^* are prone to meningioma genesis (Kalamarides et al., 2002). In contrast, conditional loss of *Nf2* using *Postn-Cre* gives rise to vestibular Schwannomas and Schwann cell hyperplasia in dorsal root ganglion (DRG) and proximal spinal nerve roots (Gehlhausen et al., 2015). However, each of these lines only forms tumors associated with that lineage and thus does not recapitulate the entire range of tumors in human NF-2 patients. Therefore, an animal model that better recapitulates the human disorder globally is still missing.

Here, we report the generation of an inducible *nf2a/b* zebrafish knockout transgenic line using the CRISPR-Cas9 approach to model NF-2. Zebrafish embryos are transparent and develop *ex utero*, easing the long-term visualization of embryos and early larvae. Zebrafish offer several advantages, including rapid development, ease of intracranial imaging and the ability to generate stable transgenic models that phenocopy human diseases, thus facilitating the study of lethal mutations. We show that *nf2a/b* is expressed in the neural crest, meninges and Schwann cells during early development and in adult Schwann cell precursors (SCPs). Loss of *nf2a/b* results in aberrant proliferation of these cell types, eventually giving rise to Schwannomas, meningiomas, cataracts and abnormal pigmentation, thus creating a useful disease model in an organism accessible to imaging and genetic manipulation at low cost.

## RESULTS

### Expression of *nf2a* and *nf2b* in the zebrafish embryo

**Fig. 1.**
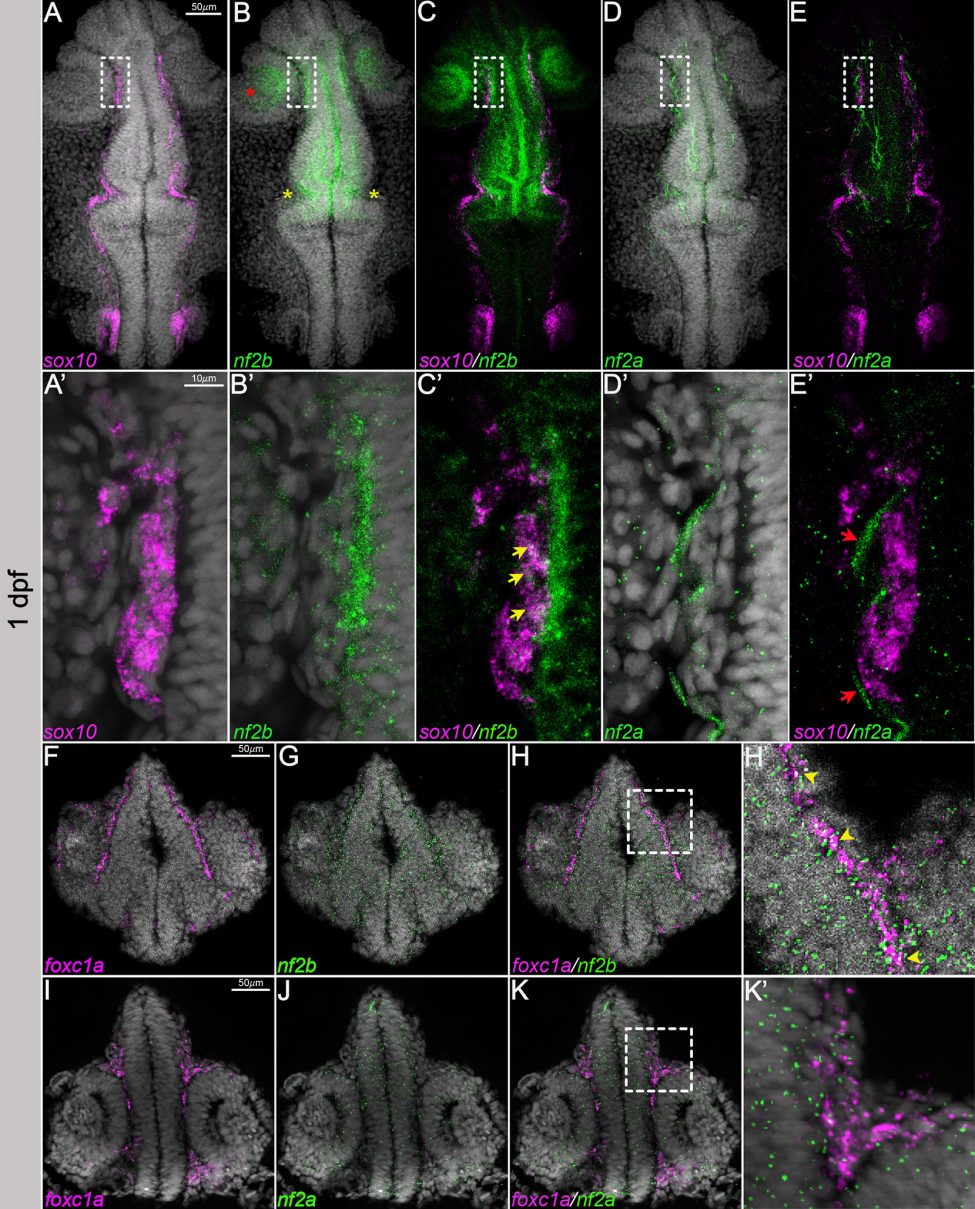
***nf2a/b* is expressed in the cranial neural crest cells and mesenchyme.** (A,B,C,D,E) Multiplexed fluorescent mRNA *in situ* hybridizations by hybridization chain reaction (HCR) reveal expression of *nf2b*, *nf2a* and *sox10* in whole-mount embryos at 1 dpf. (A′,B′,C′,D′,E′) Magnified images of the regions in the white dashed line boxes in A, B, C, D and E (yellow arrows indicate overlap of *nf2b* and *sox10* expression; red arrows indicate the ectodermal epithelial cell), (F-H,I-K) *nf2b*, *nf2a* and *foxc1a* in cryo-sectioned embryos at 1 days post fertilization (dpf). (H′,K′) Magnified images of the regions in the white dashed line boxes in H and K, respectively (yellow arrowheads indicate overlap of *nf2b* and *foxc1a* expression). Images are representative of *n*=5 embryos. DAPI (gray) was used as a counterstain. Images in A, B, C, D and E represent maximum-intensity projections of whole-mount embryos and those in A′′, B′, C′, D′ and E′ represent confocal planes of 2.3 μm thickness. F-H and I-K are histological sections of 12 μm thickness imaged with Apotome. Red asterisk indicates the optic cup; yellow asterisks indicate the midbrain–hindbrain border.

Zebrafish have two orthologs of the human *NF2* gene, *nf2a* and *nf2b*. We first examined the expression of *nf2a* and *nf2b* transcripts in 1-3 days post fertilization (dpf) zebrafish embryos using hybridization chain reaction (HCR), a highly sensitive *in situ* hybridization technique. Our results indicated that *nf2b* is the predominant paralog expressed in the early embryo at these time points. At 1 dpf, *nf2b* was broadly expressed in the cranial region, with notable signal in the forebrain, midbrain–hindbrain border, and the basal surface of the optic cup and lens. In addition, its expression overlapped with that of the neural crest marker, *sox10*, in cranial neural crest cells (Fig. 1A-C′; Fig. S1A-G). Quantification of *nf2b* and *Tg(*−7.2 *sox10:mRFP)* populations at 1 dpf indicated that ∼40% of the *sox10*-positive cells are *nf2b* positive in the cranial region (Fig. S1L). Furthermore, we observed that a few *Tg(*−7.2 *sox10:mRFP)* cranial neural crest cells were double positive for *nf2b* and *mitfa*, indicating that *nf2b* is also expressed in the melanocyte lineage (Fig. S1C,D,G), consistent with observations in mouse choroidal melanocytes (Moon et al., 2018). The expression of *nf2a* was very weak compared to that of *nf2b*, with predominant expression in epithelial cells and some overlap with *sox10* expression (Fig. 1A,D-E′). Next, we focused on the expression of *nf2a* and *nf2b* in the cranial mesenchyme. The forkhead transcription factor *Foxc1* is a crucial regulator of cranial development and is expressed in the cranial mesenchyme before skeletogenic differentiation begins and later restricted to the meningeal layers, cartilage primordium and osteoblasts (Rice et al., 2003; Vivatbutsiri et al., 2008). Zebrafish have two orthologs of *Foxc1* – *foxc1a* and *foxc1b*. *foxc1a* is expressed highly in the cranial mesenchyme at 1 dpf; *foxc1b* is expressed at later stages and labels the meningeal fibroblasts and the periocular mesenchyme (Ferre-Fernández et al., 2022; Skarie and Link, 2009). In transverse sections of the cranial region of 1 dpf embryos, we detected overlap between *foxc1a* and *nf2b* in the cranial mesenchyme (Fig. 1F-H′). In contrast to *nf2b*, *nf2a* was barely detectable at 1 dpf and displayed minimal overlap with *foxc1a* (Fig. 1I-K′).

**Fig. 2.**
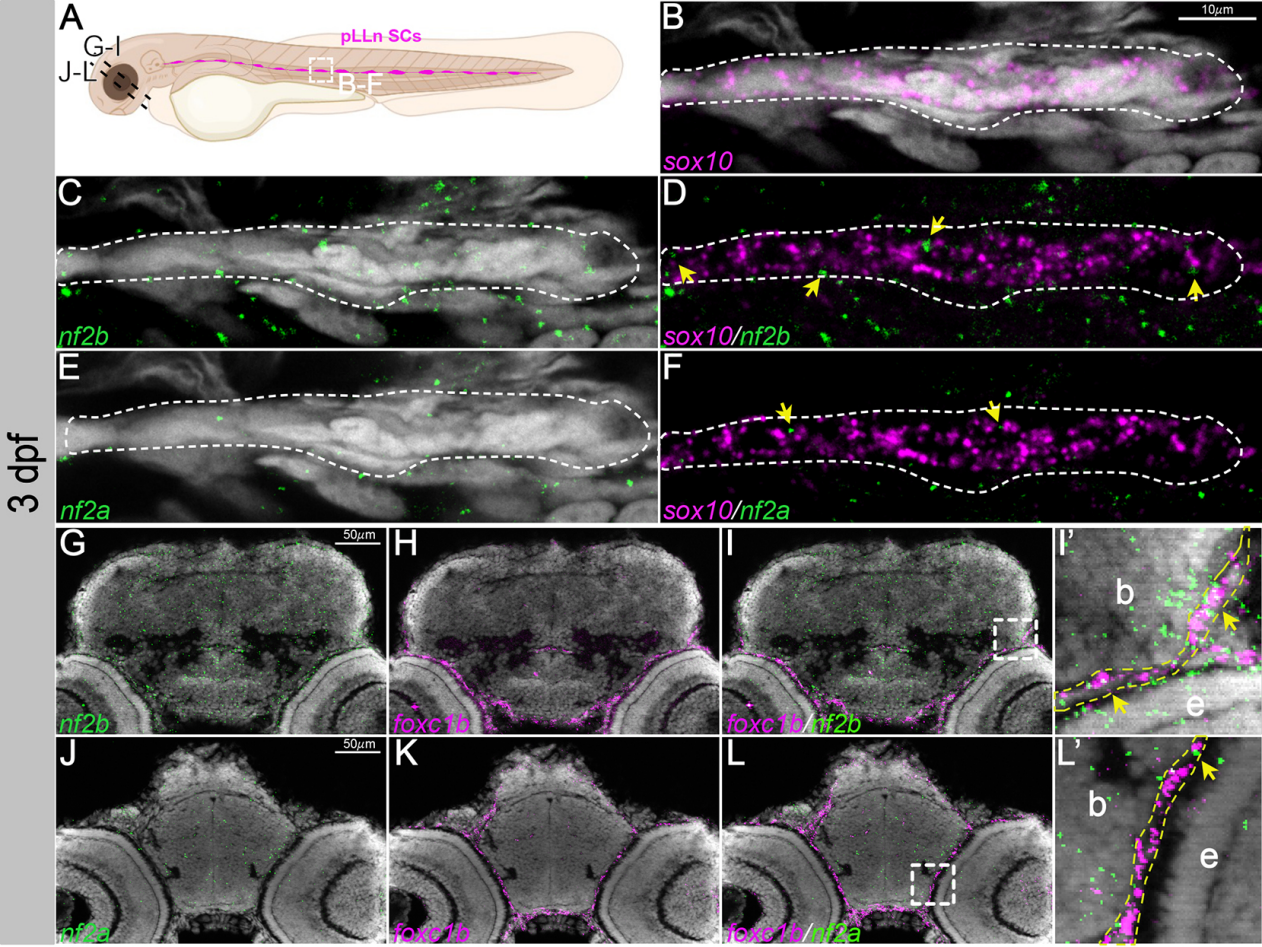
***nf2a/b* is expressed in Schwann cells and meninges.** (A) Schematic representing the posterior lateral line Schwann cells (pLLn SCs; white dashed line box) and sectioned regions (black dashed lines) at 3 dpf. (B-F) Multiplexed fluorescent mRNA *in situ* hybridizations by HCR on whole-mount embryos reveal expression of *nf2b*, *nf2a* and *sox10* in a Schwann cell cluster at 3 dpf. Yellow arrows indicate cells that have both *sox10* and *nf2* expression. White dashed lines indicate pLLn SCs expressing *sox10*. (G-I,J-L) Multiplexed fluorescent mRNA *in situ* hybridizations by HCR on cryo-sectioned embryos reveal expression of *nf2b*, *nf2a* and *foxc1b* in the cranial meninges at 3 dpf. (I′,L′) Magnified images of the regions in the white dashed line boxes in I and L, respectively. Yellow dashed lines mark the meninges layer in close proximity to the brain; yellow arrowheads indicate cells that have both *foxc1b* and *nf2* expression. Images are representative of *n*=7 embryos. Images in B-F represent confocal planes of 5 μm thickness. G-L are histological sections of 12 μm thickness imaged with Apotome. DAPI (gray) was used as a counterstain. b, brain; e, eye.

At 3 dpf, *sox10* expression is mainly observed in peripheral glia, spinal cord oligodendrocytes, and Schwann cells ranging from SCPs, immature Schwann cells, pro-myelinating Schwann cells, non-myelinating Schwann cells and myelinating Schwann cells (Wiltbank et al., 2022). At this stage, we noted several *nf2b* foci expressed in neural crest-derived Schwann cells along the posterior lateral line, labeled by *sox10* (Fig. 2A-D). Additionally, quantification of *nf2b* and *Tg(sox10:mRFP)* populations revealed that ∼45% of the *sox10*-positive posterior lateral line Schwann cells (pLLn SCs) was *nf2b* positive (Fig. S1H-K,M). We also observed a few *nf2a* foci in the Schwann cells (Fig. 2E,F). By 3 dpf, *foxc1b* was expressed in the meningeal fibroblasts and mesenchymal cells ventral to the forebrain region. We observed co-expression of both *nf2a* and *nf2b* with *foxc1b* in the meningeal fibroblasts, albeit at low levels for *nf2a* (Fig. 2G-L′).

Our results indicate that zebrafish *nf2a* and *nf2b* are expressed broadly during early development, notably in the cranial neural crest, melanoblasts, cranial mesenchyme, optic cup and lens. This is consistent with observations of *Nf2* promoter and mRNA expression in mouse embryos (Akhmametyeva et al., 2006; Huynh et al., 1996). Additionally, we detected the expression of *nf2a*/*b* in the developing Schwann cells and meningeal fibroblasts. Interestingly, *nf2b* is much more highly expressed in most tissues than *nf2a*, indicating that it is the dominant paralog. However, *nf2a* but not *nf2b* is expressed in epidermal cells, suggesting that these paralogs may also have some cell type-specific function.

### Generation of an inducible *nf2a/b* knockout line

To establish an NF-2 model, we used CRISPR-Cas9 to target zebrafish *nf2a* and *nf2b*. First, we tested guide RNAs (gRNAs) targeting both the paralogs. After determining the knockout efficiency, four gRNAs (two for each paralog) were selected for cloning into pU6x:gRNA vectors for constitutive gRNA expression. The *nf2a* and *nf2b* gRNAs containing pU6x:gRNA vectors were cloned into pGGTol2-LC-Dest-4sgRNA vector and injected into wild-type one- to two-cell zebrafish embryos with *Tol2* transposase mRNA for genomic integration. Embryos were sorted at 3 dpf for cerulean expression in the lens and grown to adulthood. Once stable F1/F2 lines were established, we used semi-quantitative RT-PCR to examine gRNA expression (Fig. S2A). As expected, we observed expression of the gRNAs in the cerulean-positive embryos but not in the cerulean-negative embryos.

**Fig. 3.**
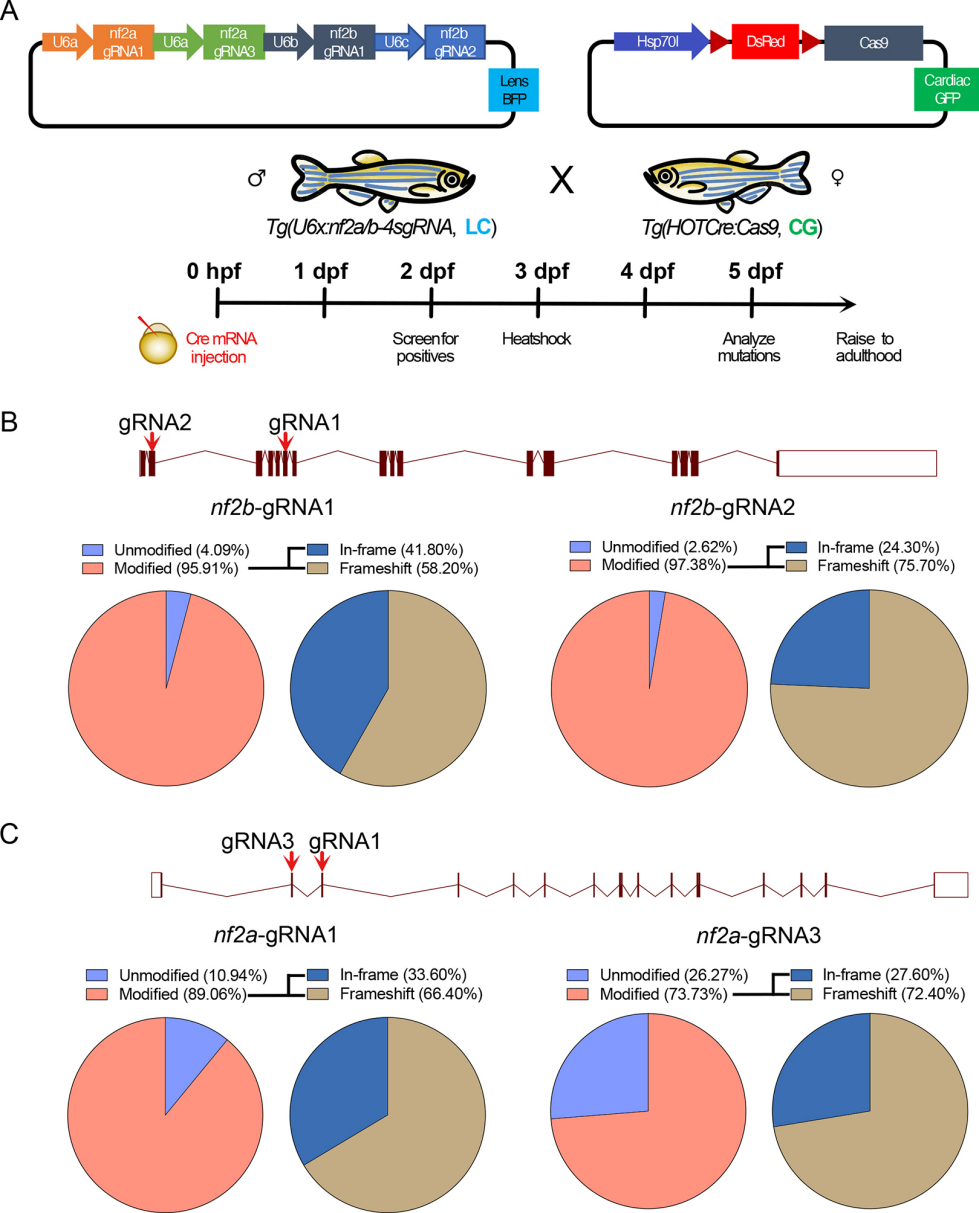
**Strategy for generation of conditional knockouts for *nf2b* and *nf2a.*** (A) Schematic of the strategy used to generate *nf2a/b* knockouts. (B,C) Schematics of target regions and pie charts represent percentages of mutated reads in *nf2b* (B) and *nf2a* (C) knockout embryos. CG, cardiac green fluorescent protein; LC, lens cerulean.

Next, to test the mutagenesis efficiency of the *Tg(pU6x:nf2a/b-4sgRNA)*-expressing stable lines, we crossed the fish with the *Tg(hsp70:loxP-mCherry-STOP-loxP-cas9)* line, referred to as *Tg(HOTCre:cas9)* (Yin et al., 2015). Injection of Cre mRNA in the progeny resulted in the removal of ‘STOP’, and heat shock induced the expression of Cas9 in the presence of the *nf2a*/*b*-targeting gRNAs (Fig. 3A illustrates the knockout strategy). Using a semi-quantitative T7 endonuclease assay, we observed that all four gRNAs could elicit mutations *in vivo* at almost 80-95% efficiency (Fig. S2B). We performed PCR sequencing for the *nf2a* and *nf2b* target regions and observed CRISPR-Cas9-induced modifications in 70-95% of the reads (Fig. 3B,C). Using western blot analysis, we observed concomitant loss of the Nf2 protein in the CRISPR-Cas9-induced animals (Fig. S2C).

Biallelic knockouts of *Nf2* mice die early during embryonic development owing to a failure to initiate gastrulation because of absence of an organized extraembryonic ectoderm (McClatchey et al., 1997). In contrast, tissue-specific *Nf2* knockouts give rise to tumors only in the associated lineages. Our strategy circumnavigates these issues by enabling tight control of the timing of Cas9 expression and the resulting mutations via heat shock to avoid disrupting *nf2a/b* at early developmental stages. We tested the survival of mosaic *nf2a/b* knockout embryos upon induction of Cas9 expression on days 1, 3 and 5 of development. Day 3 induction of Cas9 resulted in the maximum number of survivors until day 15 of development compared to day 1 and day 5 induction. Nevertheless, induction of *nf2a/b* mutation at early embryonic and larval stages primarily led to death, with ∼20-25% survivors for day 3 induction (Fig. S2D).

### Effects of *nf2a/b* knockout on neural crest derivatives, such as Schwann cells, melanocytes and meninges

**Fig. 4.**
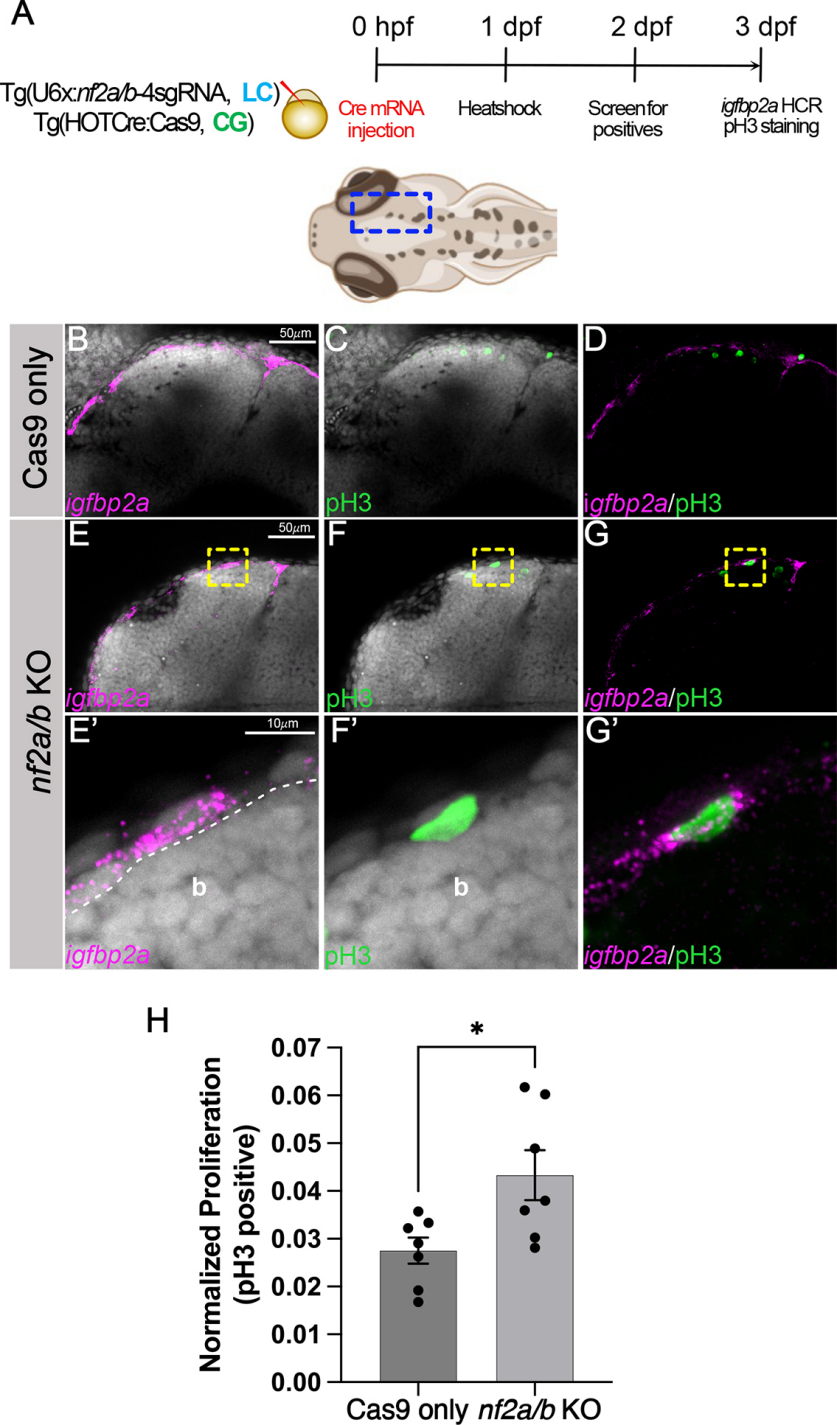
***nf2a/b* knockout leads to increased meningeal proliferation.** (A) Schematic representing the experimental strategy. Blue dashed line box in the 3 dpf zebrafish schematic indicates the imaged region. (B-G) Confocal images of the cranial region of 3 dpf Cas9-only (B-D) and *nf2a/b* knockout (E,F,G) embryos show the overlap of meningeal marker *igfbp2a* and proliferation marker pH3. (E′,F′,G′) Yellow dashed line boxes show the magnified region of the overlap in the *nf2a/b* knockout embryos. (H) Bar plot showing quantification of normalized proliferation (*P=0.0197; each dot represents data from one larva). Normalized proliferation=Igfbp2a and pH3 double-positive cells/total pH3-positive cells in the analyzed region. Each dataset was compared using unpaired two-tailed Student's *t*-test in GraphPad Prism. Error bars indicate s.e.m. across *n*=7 embryos. White dashed line marks the boundary between the brain and meninges. Images in B-E, F and G represent maximum-intensity projections of confocal planes in whole-mount embryos. E′, F′ and G′ represent confocal planes of 3.2 μm thickness. DAPI (gray) was used as a counterstain. b, brain; KO, knockout; pH3, phospho-histone 3.

**Fig. 5.**
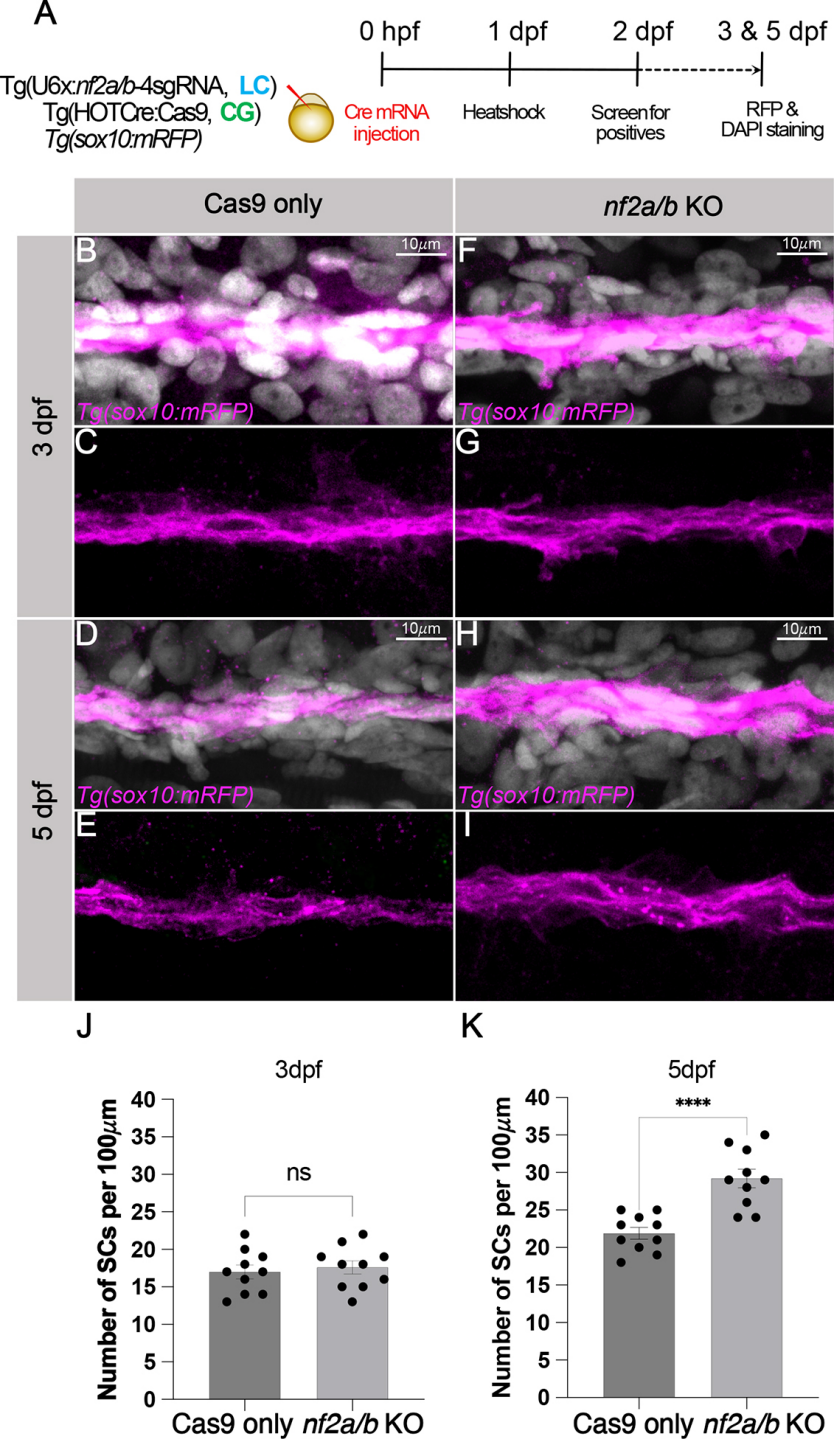
***nf2a/b* knockout leads to Schwann cell hyperplasia.** (A) Schematic representing the experimental strategy. (B-I) Confocal images of the pLLn SCs of Cas9-only controls (B-E) and *nf2a/b* knockout (F-I) embryos at 3 dpf and 5 dpf. pLLn SCs are labeled by *Tg(sox10:mRFP)* (magenta) and counterstained by DAPI (gray). Images represent confocal maximum-intensity projections of whole-mount embryos. (J,K) Bar plots showing quantification of the Sox10-positive pLLn SCs at 3 dpf (J) and 5 dpf (K) (ns, not significant; ****P<0.0001; each dot represents data from one larva). Each dataset was compared using unpaired two-tailed Student's *t*-test in GraphPad Prism. Error bars indicate s.e.m. across *n*=10 embryos. Each dot represents average of counts from three regions of pLLn SCs per embryo.

Given that zebrafish *nf2a/b* is expressed in neural crest cells, cranial mesenchyme, Schwann cells and the meninges, we tested the effect of *nf2a/b* knockout on proliferative ability in these cell types. First, we compared meningeal cell proliferation at 3 dpf between Cas9-only controls and *nf2a/b* conditional knockouts (heat shock at 1 dpf) (Fig. 4A). We used *igfbp2a* as a marker to label the meninges and found increased proliferation in the meningeal cells after *igfbp2a* knockout (Fig. 4B-G′). Quantitation of this effect demonstrated a significant increase in meningeal phospho-histone 3 (pH3) staining after the loss of *nf2* (Fig. 4H). Next, we utilized the transgenic line *Tg(*−7.2 *sox10:mRFP)* to visualize Schwann cells along the posterior lateral line at 3 dpf and 5 dpf. Although we did not see any difference in the Schwann cell number at 3 dpf between *nf2a/b* knockouts and Cas9-only controls (Fig. 5A,B,C,F,G,J), we observed a striking increase in the numbers and thickness of Schwann cell clusters along the posterior lateral line in *nf2a/b* knockout larvae compared to the Cas9-only controls (Fig. 5D,E,H,I,K) at 5 dpf, an observation strikingly similar to that seen in zebrafish *nf1* knockouts (Shin et al., 2012).

**Fig. 6.**
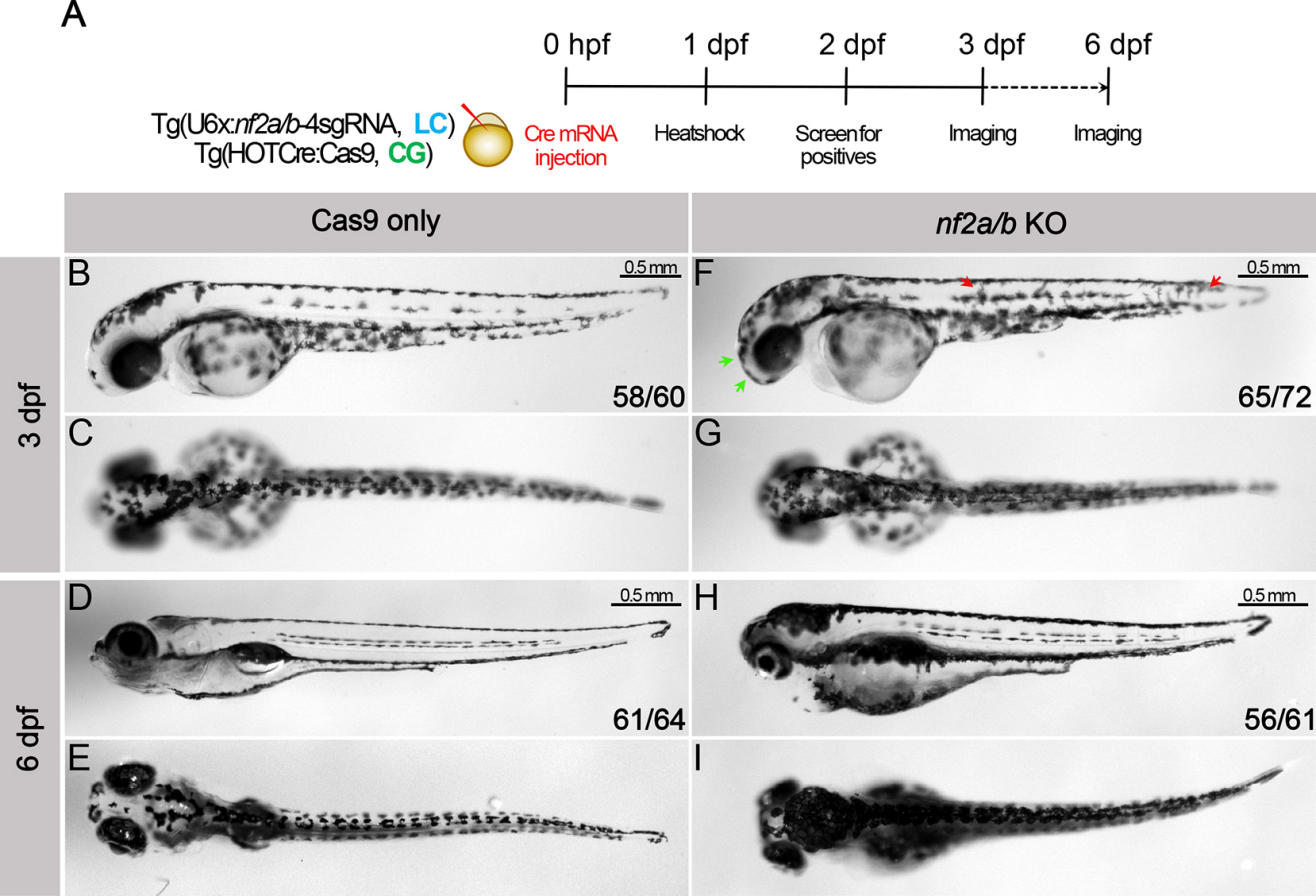
**Knockout of *nf2a/b* leads to hyperpigmentation*.*** (A) Schematic representing the experimental strategy. (B-I) Lateral and dorsal bright-field images of Cas9-only control (B-E) and *nf2a/b* knockout (F-I) embryos at 3 dpf and 6 dpf. Green arrows indicate increased melanophores in cranial region; red arrows indicate migratory melanophores. Numbers represent phenotypes across *n*=3 independent experiments.

NF-2 patients frequently present with cafe-au-lait macules, which are hyperpigmented regions in the skin. Consistent with this, we observed striking hyperpigmentation in *nf2a/b* knockouts compared with Cas9-only controls at 3 dpf (Fig. 6A,B,C,F,G). At this stage, most of the Cas9-only control melanocytes were settled in the dorsal/midline/ventral pigmentation pattern; however, we noted that melanocytes in *nf2a/b* knockouts were not completely patterned and appeared to still be migratory (Fig. 6F, red arrows). In addition, we observed a marked increase in melanocyte density in the cranial regions (Fig. 6F, green arrows). At 6 dpf, we observed dramatic hyperpigmentation of the head, reduced body length, small eyes and impaired inflation of the swim bladder in *nf2a/b* knockouts (Fig. 6D,E,H,I). Taken together, the results suggest that the loss of *nf2a/b* in zebrafish affects many of the same cell types that are prone to tumorigenesis in human NF-2 patients.

### Tumor formation in adult *nf2a/b* knockout zebrafish

**Fig. 7.**
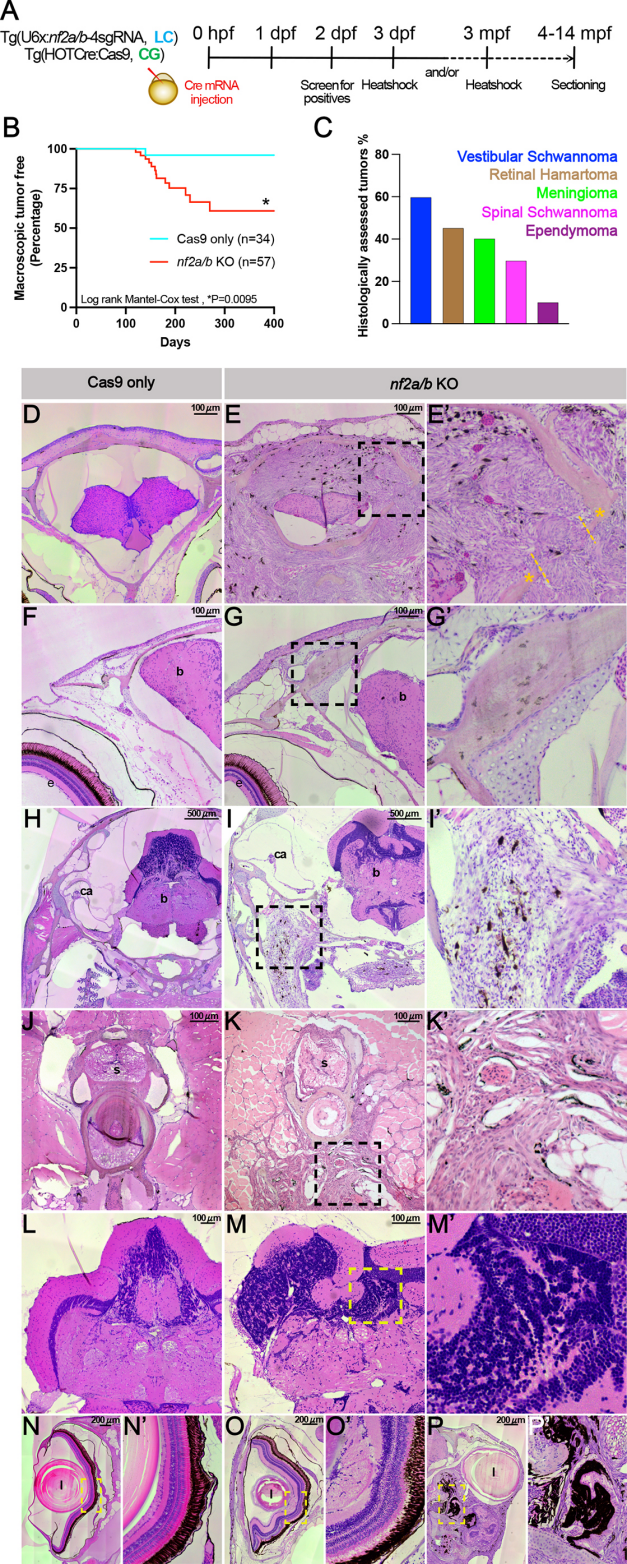
***nf2a/b* knockout leads to tumors in adult zebrafish*.*** (A) Schematic representing the experimental strategy. (B,C) Graphs representing tumor-free survival in Cas9-only and *nf2a/b* knockout fishes (B) (log rank Mantel–Cox test; *P=0.0095; performed using GraphPad Prism) and histological assessment of tumors (C). Histological assessment was performed for *n*=12 fish and represents the overall incidence of each tumor type. Few fish developed multiple tumors. (E-P′) Transverse sections of *nf2a/b* knockout adult zebrafish show meningioma (E,E′), skull bone thickening (G,G′), vestibular Schwannoma (I,I′), trunk Schwannoma (K,K′), ependymoma (M,M′), epiretinal membrane (O,O′) and retinal hamartoma (P,P′). Cas9-only sections (D,F,H,J,L,N) are shown for comparison. Black and yellow dashed line boxes outline the zoomed regions. The majority of the tumor samples display abundant melanocytes within the tumors. b, brain; ca, crista anterior; e, eye; l, lens; s, spinal cord. Yellow asterisks indicate the cranium; yellow dashed lines indicate meningioma penetrating the cranium.

Given that most embryos die after heat shock-mediated Cas9 induction during the first few days of development, for long-term survival that would allow analysis of tumor formation, induction was conducted in 3- to 6-month-old fish. Zebrafish were allowed to develop 1-10 months after the induction. At these time points, we noted issues with balance, cataracts, hyperpigmented regions and conspicuous tumors developing 1-6 months after induction (Fig. 7A,B). Interestingly, loss of balance and issues with swimming were some of the first phenotypic manifestations observed in the *nf2a/b* knockouts, indicating vestibular dysfunction.

To characterize tumor morphology, adult fish were fixed and sectioned in the transverse plane. Sections were stained with Hematoxylin/Eosin and examined at the brain or spinal cord level and surrounding tissues. Quantification of histological sections of Cas9-only controls and *nf2a/b* knockouts revealed tumors of several subtypes. Twelve fish were analyzed, some of which had multiple tumor types. Tumors primarily consisted of vestibular (60%) and spinal Schwannomas (30%), meningiomas (40%), retinal hamartomas (45%) and ependymomas (10%) (Fig. 7C). In Cas9-only control animals, we did not observe any aberrant growth of the involved tissues (Fig. 7D,F,H,J,L,N). In the *nf2a/b* knockouts, we observed transitional meningiomas (containing fibroblastic and meningothelial components). In addition, some of these meningiomas penetrated the cranium and appeared metastatic (Fig. 7E,E′). When focusing on the cranium, the Cas9-only control fish appeared normal, whereas the *nf2a/b* knockouts displayed thickening of the bones (Fig. 7G,G′) (Li et al., 2018).

Bilateral vestibular Schwannomas are one of the primary diagnostic features of NF-2. Although the auditory nerves of controls appeared normal (Fig. 7F), by contrast, vestibular Schwannomas were observed in the *nf2a/b* knockout animals (Fig. 7I,I′). At the spinal cord level, we noted Schwannoma-like tumors that appeared to have aggressively metastasized into muscle tissues in the *nf2a/b* knockouts (Fig. 7K,K′). We also observed cranial ependymomas (Fig. 7M,M′). Finally, we looked at the sections of the eye in the Cas9-only controls, which were arranged into distinct retinal layers similar to the human retina (Fig. 7N,N′) (Richardson et al., 2017). The *nf2a/b* knockouts displayed a distorted morphology, with epiretinal membranes and loss of distinct layers (Fig. 7O,O′). Additionally, we observed some knockouts with severe retinal hamartomas (Fig. 7P,P′).

Although the tumors arising in NF-2 seem to be in different anatomical locations, one likely cell of common origin for NF-2 tumors is SCPs. These neural crest-derived cells persist along peripheral nerves through adulthood and can migrate, differentiate and dedifferentiate under appropriate conditions. Although they are able to form Schwann cells, SCPs are often multipotent and can differentiate into pigment cells and a wide range of neural crest-derived cell types (Solovieva and Bronner, 2021). Their broad developmental potential correlates with the broad range of tumor phenotypes in NF-2 patients, including spinal, peripheral and cranial nerve tumors (Gehlhausen et al., 2015). To ascertain whether *nf2a/b* is expressed in SCPs, we performed HCR in 3-month-old zebrafish sections and observed *nf2b* mRNA expression in ventrolaterally positioned DRG-associated SCPs labeled by *sox10* (Kamenev et al., 2021) (Fig. S3).

Ideally, we would like to recognize tumors in a non-invasive manner. To this end, we subjected 5- to 12-month-old Cas9-only control and *nf2a/b* knockout animals to analysis by optical coherence tomography (OCT) (Fig. S4). The results demonstrated increased telencephalic ventricular volume in the *nf2a/b* knockouts compared with Cas9-only controls. A possible explanation for this is that the tumors obstruct cerebrospinal fluid flow, resulting in an enlarged intraventricular space that can be detected in the intact animal. This is consistent with observations of hydrocephalus resulting from cerebrospinal fluid blockage in some NF-2 patients (Tanrıkulu and Özek, 2019).

## DISCUSSION

NF-2 is an inherited condition that increases the risk of developing specific nervous system tumors such as bilateral vestibular Schwannomas, multiple spinal and peripheral Schwannomas, meningiomas and ependymomas. NF-2 is caused by inactivating variants in the *NF2* gene, which may be germline or somatic. Two clinical forms of NF-2 have been historically documented. The Wishart phenotype represents a more aggressive manifestation of the condition, characterized by the development of multiple neoplasms in patients under 20 years old, with rapid progression of lesions. On the other hand, some patients display a less severe phenotype known as the Gardner phenotype, characterized by fewer slow-growing tumors that typically appear later in life. It is now understood that the specific type of alteration in the *NF2* gene primarily influences the severity of the disease spectrum. Patients with truncating alterations that deactivate *NF2* tend to experience a more severe disease course, whereas those with missense loss-of-function variants generally have a milder disease progression (Halliday et al., 2017).

To date, existing mouse models for NF-2 have failed to fully recapitulate the human phenotype. Although homozygous loss of *Nf2* causes early mortality (McClatchey et al., 1997), hemizygous or heterozygous loss of *Nf2* results in a tumor spectrum different from that seen in the human counterparts (McClatchey et al., 1998). Tissue-specific conditional knockouts produce tumors in select tissues rather than the whole range of tumors found in patients (Gehlhausen et al., 2015; Giovannini et al., 2000). In recent years, the zebrafish has become a robust model organism for cancer research owing to rapid embryonic development, high fecundity, amenability to genetic manipulation, drug treatments and transparency throughout early developmental stages, allowing for powerful *in vivo* imaging (Patton et al., 2021; Roy et al., 2024; Shin et al., 2012; White et al., 2013). Moreover, the results obtained from zebrafish models can be translated back to humans owing to the highly conserved nature of these cancer-related programs. Thus, the zebrafish serves as a non-mammalian vertebrate organism that represents a cost-effective model to study the effects of loss of genes like *NF2* on tumor formation.

Our study found a broad range of phenotypes in 4- to 13-month-old zebrafish after mutagenesis of *nf2a/b* in 3-month-old fish – these include meningiomas, vestibular Schwannomas, cataracts, retinal hamartomas and ependymomas. Although many animal models require the knockout of a gene in a sensitized background (Shin et al., 2012), we find that the *nf2a/b* mutations alone appear sufficient in wild-type fish to produce tumors. A likely explanation for these observations is the inducible nature of *nf2a/b* mutagenesis, wherein we can elicit biallelic mutations (a prerequisite for NF-2-related tumorigenesis) at the desired time of the animal's lifespan. We demonstrate that zebrafish *nf2a/b* is expressed in developing cell types, such as cranial neural crest, melanoblasts, meningeal fibroblasts, SCPs and Schwann cells, all later associated with NF-2 tumors. Additionally, the knockout of *nf2a/b* during early development results in the hyperproliferation of these cell types.

A puzzling observation from our study and earlier work is that even though *NF2* is widely expressed during embryonic development, its inactivation predisposes tumors only in specific tissues. According to Knudson's two-hit hypothesis, it is believed that NF-2-associated tumors occur owing to additional somatic genetic alterations in susceptible cell populations, leading to the biallelic loss of function of *NF2* (Woods et al., 2003). Another likely explanation could be the mechanosensitivity of the involved tissue types. The tumors prevalent in NF-2 – meningioma, Schwannoma, retinal hamartoma, etc. – arise from precursor cell types such as meningeal fibroblasts, Schwann cells and retinal cells, respectively. These cells are known to be present as stratified layers (e.g. meninges and retina) or tightly woven in peripheral nerves (e.g. Schwann cells). Such arrangement can lead to changes in the nuclear to cytoplasmic connections and facilitate the aberrant transport of nuclear effector/oncogenes in the *NF2* variant background. One such example is the protein YAP (also known as YAP1), which is known to aid *NF2-*mediated tumorigenesis (Guerrant et al., 2016; Laraba et al., 2022; Oh et al., 2015; Szulzewsky et al., 2022). Although several studies have explored the functional role of YAP in combination with merlin in tumorigenesis, the mechanobiological aspect of their interaction has yet to be decoded. Recently, Alasaadi et al. (2024) demonstrated the role of YAP in neural crest competency via hydrostatic pressure. Thus, it is possible that tissue mechanics interplay with signaling pathways to regulate tumor induction in NF-2.

In summary, we describe a new zebrafish model for conditional inactivation of *nf2a/b*. Because our inducible model can undergo conditional inactivation of *nf2a/b* over various ages, this model can potentially recapitulate neurofibromatosis onset at different stages of life. Moreover, it holds the promise of enabling testing of therapeutic agents or large-scale chemical libraries for the ability to ameliorate the phenotypes of NF-2. The results demonstrate the utility of this model for recapitulating a broad range of phenotypes associated with NF-2 that closely mimic human disease. The accessibility, ease of manipulation, availability of genetics and facility of imaging promise to make this an extremely useful model for further exploration of tumor ontogeny and assaying means of treatment.

## MATERIALS AND METHODS

### Zebrafish lines

Zebrafish (*Danio rerio*) were maintained at 28°C, with adults on a 14 h light/10 h dark cycle. All zebrafish work complied with the California Institute of Technology (Caltech) Institutional Animal Care and Use Committee (IACUC). Transgenic lines used in this study were the ABWT (Zebrafish International Resource Center), *Tg(HOTCre:Cas9)* (a kind gift from Dr Wenbiao Chen, Vanderbilt University School of Medicine, Nashville, TN, USA), *Tg(−7.2kb-sox10:mRFP)* and *Tg(U6x:nf2a/b-4sgRNA)* (generated at the Bronner laboratory, Caltech) lines.

### HCR and immunohistochemistry

HCR v3.0 was performed according to the zebrafish protocol suggested by Molecular Technologies with several modifications. Briefly, methanol-fixed embryos were rehydrated by a series of methanol/PBS–Tween-20 solutions (2×100%, 75%, 50%, 25%; every step 15 min), washed in PBS–Tween-20 (2×10 min), depigmented by a bleaching mix solution (formamide, 20× SSC, 30% hydrogen peroxide and distilled water) under direct light, washed in PBS–Tween-20 (10 min), treated with proteinase-K (20 μg/ml) according to the age of the larvae, washed in PBS-Tween-20 (2×10 min), post-fixed in 4% paraformaldehyde (PFA) (20 min), washed in PBS–Tween-20 (3×10 min), pre-hybridized in 30% probe hybridization buffer at 37°C (60 min), and incubated with probes (2-4 μl of 2 μM stock per probe mixture) in probe hybridization buffer at 37°C overnight. All probes, hairpins and buffers were designed and ordered through Molecular Technologies. The samples for histological analysis were embedded in agarose/Tissue-Tek O.C.T. compound (Sakura Finetek, USA), sectioned (12 μm for embryo and larvae) and counterstained with 4′,6-diamidino-2-phenylindole (DAPI).

pH3 immunostaining was performed on either HCR-processed or methanol-fixed embryos for proliferation assays. Briefly, HCR-processed embryos were transferred to 1× PBS–Triton X-100 (3×10 min), or methanol-fixed embryos were rehydrated by a series of methanol/PBS–Triton X-100 solutions (2×100%, 75%, 50%, 25%; every step 15 min), blocked with 10% donkey serum–1× PBS–Triton X-100 for 4 h at room temperature. The embryos were incubated in anti-pH3 antibody overnight at 4°C. The next day, the samples were washed in PBS–Triton-X100 (4×30 min) at room temperature and incubated in a secondary antibody (Thermo Fisher Scientific, A21202) for 4 h at room temperature. The samples were then rewashed in PBS–Triton X-100 (4×30 min) at room temperature. All whole-mount images were imaged with a Zeiss LSM 900 confocal microscope.

For sectioning, we euthanized fish at 3 months post fertilization according to Caltech IACUC, followed by fixation with 4% PFA overnight, three once-per-hour washes with 1× PBSTw (1× PBS+0.1% Tween-20) and serial methanol fixation (25%, 50%, 75%, 2×100%) and stored at −20C overnight. Next, methanol-fixed fish were rehydrated by a series of methanol/PBS–Tween-20 solutions (2×100%, 75%, 50%, 25%; every step 15 min), washed in PBS–Tween-20 (2×10 min) and treated in 30% sucrose for 24 h, embedding in Tissue-Tek O.C.T. compound (Sakura Finetek, USA), and preparation of 16 μm and 50 μm sections using a Cryostat HM525 NX. HCR on sections was performed according to Stundl et al. (2023). Briefly, sections were treated with 10 µg/ml proteinase K at 37°C for 10 min, rinsed with 1× PBSTw and hybridized overnight at 37°C with 5 μl of 1 μM probe stock/300 μl hybridization solution. Anti-Sox10 antibody staining on HCR-processed sections was performed according to Kamenev et al. (2021). All histological section images were acquired with a Zeiss AxioImager.M2 equipped with an Apotome.2.

Quantifications for meningeal proliferation (Fig. 4H), Schwann cell hyperplasia (Fig. 5J,K), and *nf2b* HCR signal in cranial neural crest cells and pLLn SCs (Fig. S1L, M) were performed using ImageJ cell counter feature by manually labeling the cells. The data were plotted using GraphPad Prism software.

### CRISPR-Cas9 strategy

gRNAs for zebrafish *nf2a* and *nf2b* (Table S1) were designed using CHOP-CHOP. gRNAs targeting zebrafish *nf2a/b* were validated and cloned in the pU6(x):sgRNA#(x) vectors. All four guides were cloned into the final destination vector pGGDestTol2LC-4sgRNA (*nf2a/b*-4sgRNA). The pU6(x):*nf2a/b*-4sgRNA vector and Tol2 transposase mRNA were injected into ABWT one- to two-cell embryos. Embryos were sorted at 3 dpf for cerulean expression in the lens and grown to adulthood. Two independent stable lines were established for the pU6(x):*nf2a/b*-4sgRNA transgene (Fig. S1A,B). For generating *nf2a/b* knockouts, *Tg(U6x:nf2a/b-4sgRNA)* fish were crossed with *Tg(hsp70:loxP-mCherry-STOP-loxP-cas9)*, referred to as *Tg(HOTCre:Cas9)* fish. Then, 10-20 pg Cre recombinase mRNA was injected into the embryo. Heat shock was performed by adding 40°C E3 water, and incubation at 38°C for 15 min and 30 min was applied to embryos or adults, respectively, at the indicated stages.

### T7 endonuclease assay and premium PCR sequencing

Genomic DNA was isolated from control and *nf2a/b* knockout embryos following the protocol described in The Zebrafish Book. gRNA target regions for *nf2a* and *nf2b* were amplified using the primers listed in Table S1. T7 endonuclease reaction was set up according to Lingeman (2017). The digested products were then electrophoresed in a 2% agarose gel. Purified PCR products were sent for premium PCR sequencing to Primordium Labs. At least 10,000 high-quality reads were analyzed for each sample using CRISPResso2 (Clement et al., 2019).

### RNA isolation, cDNA synthesis and PCR

RNA was isolated from zebrafish embryos using Nucleospin Triprep (Macherey Nagel, 740966) according to the manufacturer's protocols. cDNA was synthesized using a Superscript III First-Strand cDNA Synthesis Kit (Thermo Fisher Scientific, 1800051). PCR was carried out using the primers listed in Table S1. PCR products were electrophoresed in a 1.5% agarose gel.

### Protein extraction and western blotting

Zebrafish embryos were dechorionated and deyolked in an ice-cold ringer solution. Approximately 100 embryo bodies were resuspended in 50 μl NP40 lysis buffer (Thermo Fisher Scientific, FNN0021). The NP40 lysis buffer was supplemented with a 1× protease inhibitor cocktail (Sigma-Aldrich, 05892970001). The embryo bodies were crushed using a pestle homogenizer and kept on ice for 15 min. The embryo bodies were homogenized again and kept on ice for another 15 min. The sample was centrifuged at 13,800 ***g*** for 20 min at 4°C. The soluble fraction of cell lysate was collected in a fresh tube. The protein concentration in the soluble fraction was quantified using a bicinchoninic acid protein estimation kit (Thermo Fisher Scientific) using known concentrations of bovine serum albumin as standard. Then, 30-50 μg of the protein was run on 10% SDS-PAGE gels. Proteins were separated by using 25 mA per gel in the electrophoresis buffer. The resolved samples were transferred to 0.2um Immobilon-FL PVDF membranes (Millipore) (activated in absolute methanol for 30 s) using wet transfer (in transfer buffer containing 14.4 g glycine, 3.03 g Tris and 20% methanol). The transfer was carried out for 90 min at 300 mA at 4°C, and membranes were blocked with 5% skim milk in 0.1% Tris-buffered saline with Tween-20 (TBST) for 1 h at room temperature. Subsequently, the blot was incubated with a 1:1000 dilution of anti-merlin antibody overnight at 4°C. Subsequent to incubation with the primary antibody, the membrane was washed with 0.1% TBST and incubated with the corresponding secondary antibody conjugated to horseradish peroxidase (GE Life Sciences, NA934) at 1:10,000 dilution for 1 h at room temperature. The membrane was washed with 0.1% TBST and developed using ECL reagent (Millipore, WBKLS0500). Immunoblots were imaged using conventional chemiluminescent immunoblotting.

### OCT imaging

OCT imaging of the zebrafish brain was conducted using a custom OCT system, with details of the system described in a previous paper (Kim et al., 2019). Briefly, the OCT system comprises a swept source operating at 1310 nm, offering a bandwidth of ∼93 nm and a sweep rate of 100 kHz. This setup affords an axial resolution of ∼18 μm (in air) and a lateral resolution of ∼30 μm. The euthanized zebrafish were placed in a Petri dish with a foam holder to secure their position. The custom OCT system, integrated with a camera and an indicator laser, assisted in determining the imaging position of the zebrafish. Three-dimensional OCT images were acquired, covering an imaging area of 9×9 mm.

### Zebrafish adult head histology

The adult zebrafish heads for histological analysis were prepared as described in Stundl et al. (2023). Briefly, the samples were rinsed in distilled water, decalcified in Morse's solution and embedded into the JB4 resin [prepared according to the manufacturer (Sigma-Aldrich)’s instructions] at room temperature overnight. The next day, the infiltration solution was replaced by an embedding solution (prepared according to the manufacturer's instructions), placed into an embedding mold (Polyscience) and transferred to a vacuum chamber, which accelerated the polymerization (∼3 h). The resin block was sectioned at 7 μm, and sections were stained with Mayer's Hematoxylin.

## Supplementary Material

10.1242/dmm.050862_sup1 Supplementary information
