# Supplementary material for: Generation of a zebrafish neurofibromatosis model via inducible knockout of nf2a/b
Source: Dis Model Mech. 2024 Dec 4;17(12):dmm050862. doi: 10.1242/dmm.050862 (PMC11646113; doi:10.1242/dmm.050862)
Supplement: Supplementary information [file dmm-17-050862-s1.pdf]

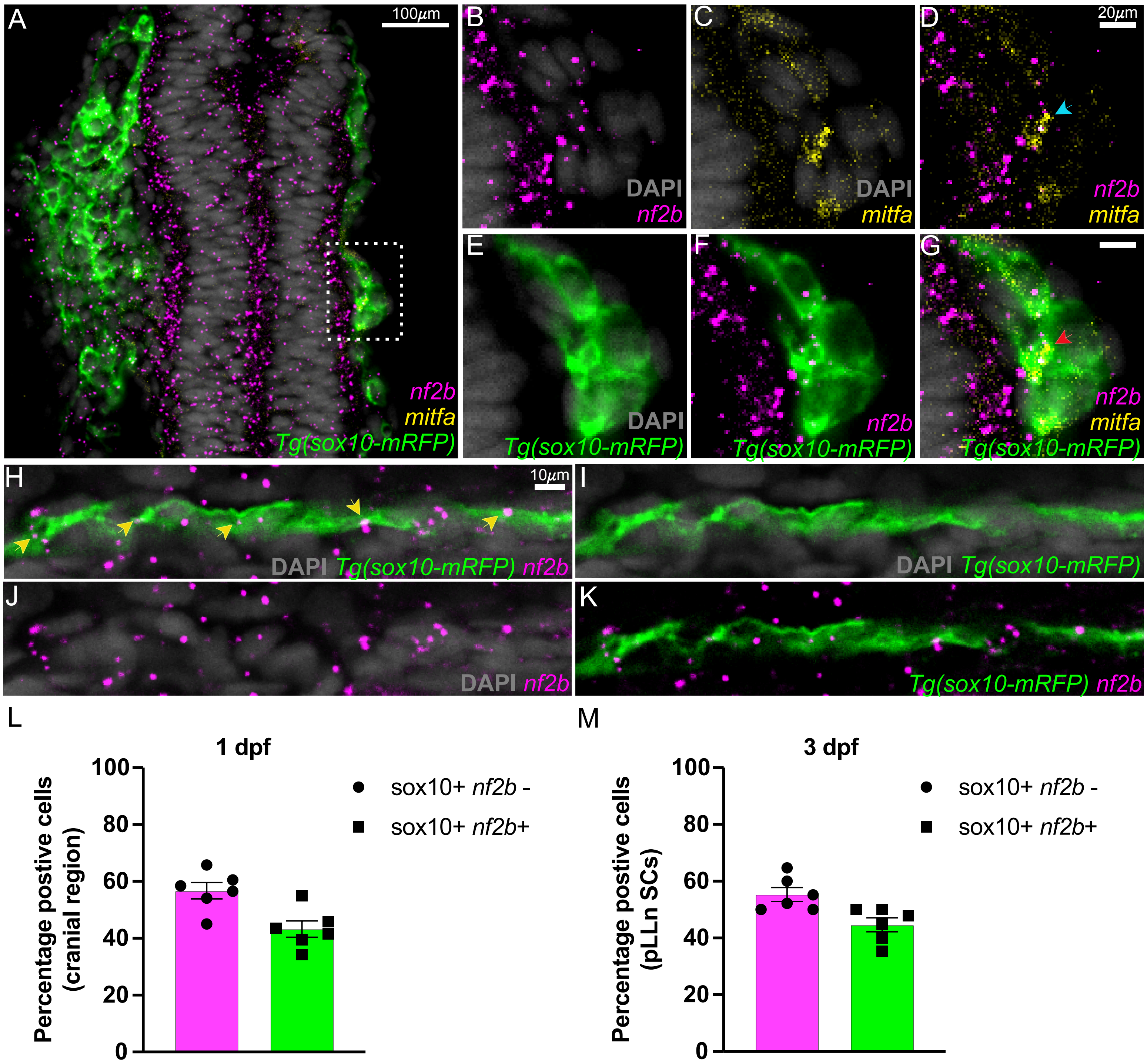

Fig S1. *nf2b* is expressed in *Tg(-7.2 sox10:mRFP)* positive cranial neural crest cells and pLLN Schwann cells. Multiplexed fluorescent mRNA in situ hybridizations by HCR reveals expression of (A-G) *nf2b* in *Tg(-7.2 sox10:mRFP)* positive cells at 1 dpf. (B-G) represent zoomed image of regions in dotted white box (cyan arrow represent overlap of *nf2b* and mitfa expression, red arrow represents overlap of *nf2b* and *mitfa* in *Tg(-7.2 sox10:mRFP)* postive cell). (H-K) represent *nf2b* expression in pLLN Schwann Cells labelled by sox10:mRFP (yellow arrow represent overlap of *nf2b* and *Tg(sox10:mRFP)* expression). Images represent confocal planes of 1.4µm thickness. Graphs representing quantifications of sox10+/*nf2b*- and sox10+/*nf2b*+ cells at (L) 1 dpf cranial region and (M) 3 dpf pLLN SCs across N=6 embryos (each dot represents data of one embryo). Scale bar in (A) 100µm. Scale bar in (B-G) 20µm. Scale bar in (H-K) 10µm.

Disease Models & Mechanisms • Supplementary information

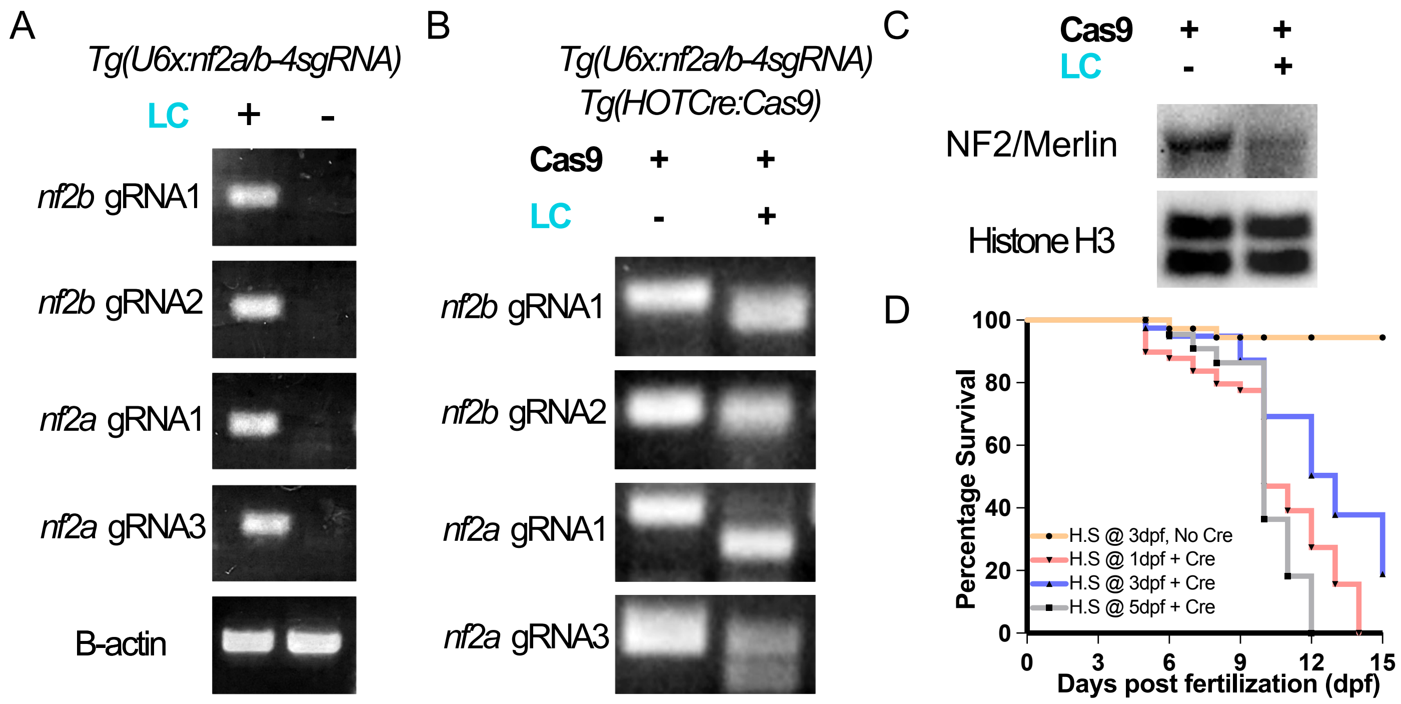

Fig. S2. Validation of stable transgenic line *Tg(U6x:nf2a/b-4sgRNA)::HOTCre: Cas9.* (A) Agarose gel images showing the expression of the guide RNAs in the stable transgenic line. (B) Agarose gel image of T7 endonuclease assay for *nf2a/b* guide RNA target regions. (C) Western blot image showing down regulation of NF2 protein in *nf2* knockouts, Histone H3 was used as the loading control. (D) Survival plot of *Tg(U6x:nf2-4sgRNA):HOTCre:Cas9* after heatshock in the presence/ absence of Cre-recombinase mRNA at different embryonic/larval stages. LC + indicates lens cerulean positive (contains *nf2a/b* targeting guide RNAs) and Cas9 + indicates cardiac GFP positive (expresses Cas9 upon heatshock) embryos were used for the experiment.

Disease Models & Mechanisms • Supplementary information

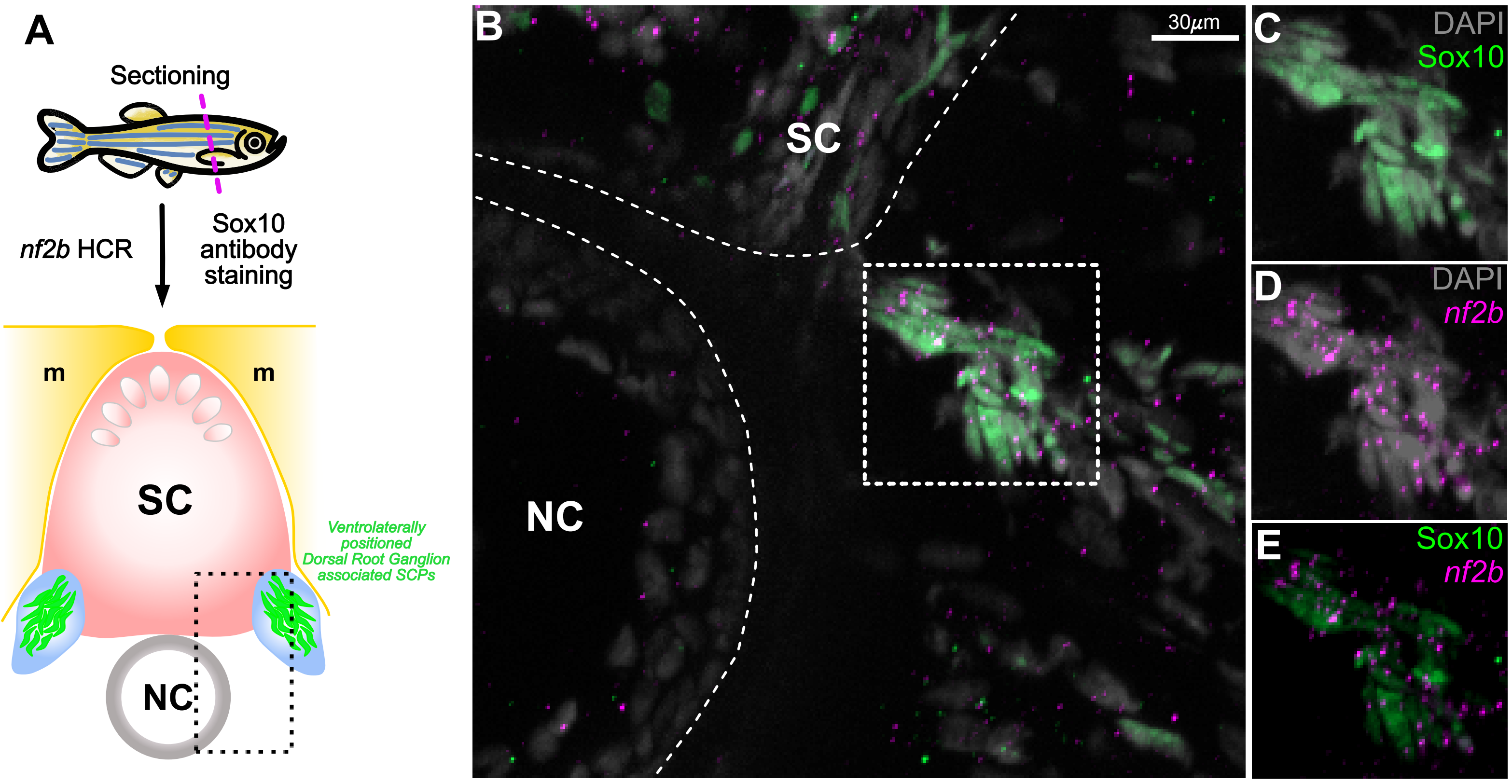

Fig. S3. ***nf2b*** **is expressed in Schwann Cell Precursors (SCPs).** (A) Schematic illustration of experimental strategy in 3 month old (mpf) zebrafish. Dotted red box represents the analyzed region. (B)Fluorescent mRNA in situ hybridizations by HCR coupled with antibody staining reveals expression of *nf2b* in SCPs labelled by Sox10 antibody. White dotted curves represent boundaries of SC and NC. White dotted box represents the area highlighted in (C-E). Scale bar in (B) 30μm. SC- Spinal Cord, NC - Notochord, m - Muscle.

Disease Models & Mechanisms • Supplementary information

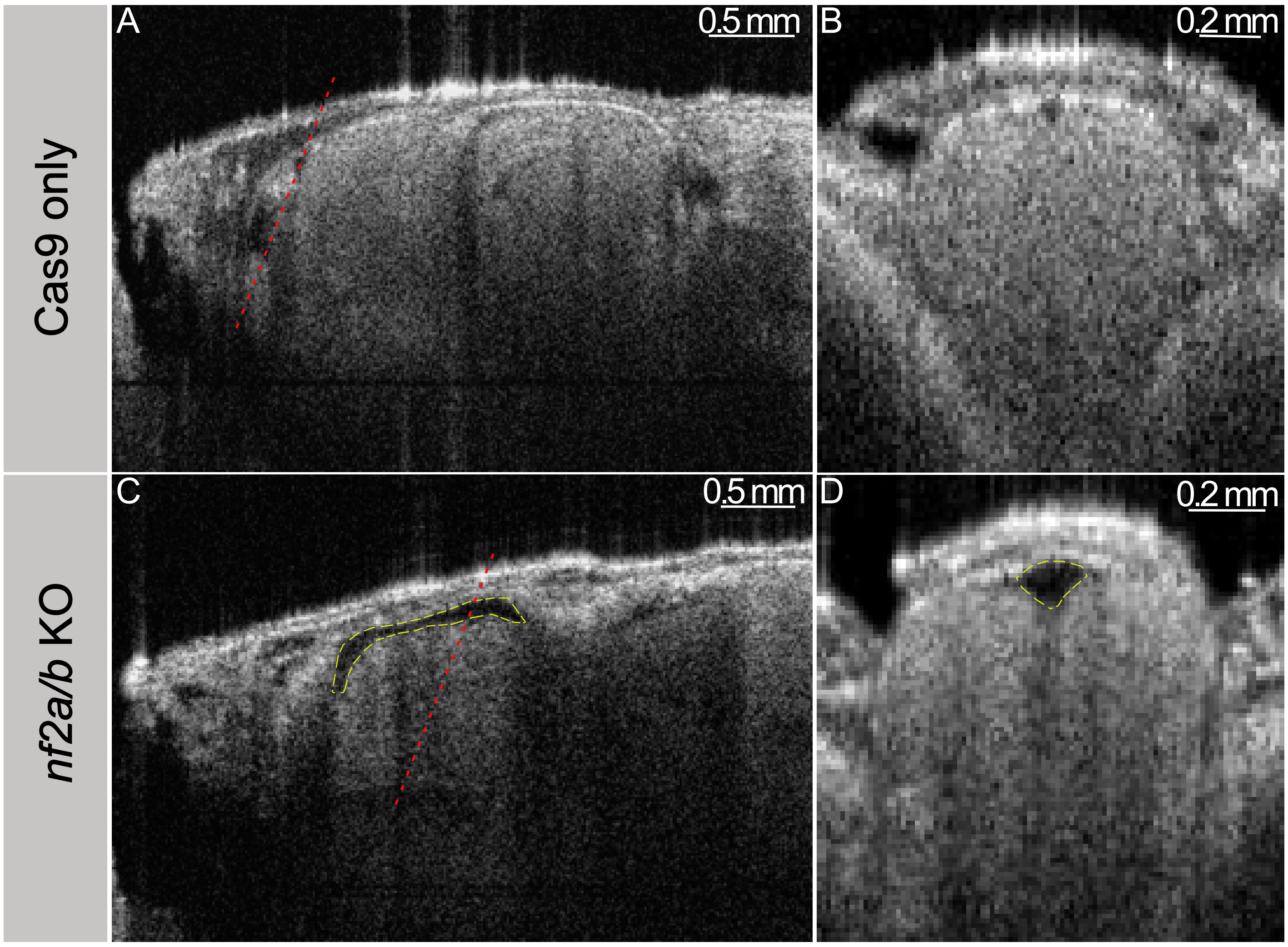

Fig. S4. *nf2a/b* knockout adult zebrafish display enlarged telencephalic ventricles. OCT images of euthanized adult zebrafish (12mpf) revealed enlarged telencephalic ventricles (highlighted with dashed yellow line) in *nf2a/b* knockout (C,D) animals as compared to Cas9-only controls (A-B). Transverse section taken as indicated by red dashed line.

Table S1. List of reagents used in this study

Reagent or Resource	Source, Identifier
**Experimental Models: Organisms/Strains**	
ABWT	ZIRC, ZDB-GENO-960809-7
Tg(-7.2sox10:mRFP)	ZDB-TGCONSTRCT-080321-2
Tg(pU6x:*nf2a/b*-4sgRNA)	This paper
Tg(HOTCre:Cas9)	ZDB-TGCONSTRCT-170418-5
**Plasmids**	
pCS2-Cre.zf1	Addgene; Plasmid #61391
pGGDestTol2LC-*nf2a/b*-4sgRNA	This paper; modified from addgene plasmid Plasmid #64242
pU6a-*nf2a*-gRNA1	This paper; modified from addgene plasmid Plasmid #64245
pU6a-*nf2a*-gRNA3	This paper; modified from addgene plasmid Plasmid #64246
pU6b-*nf2b*-gRNA1	This paper; modified from addgene plasmid Plasmid #64247
pU6c-*nf2b*-gRNA2	This paper; modified from addgene plasmid Plasmid #642428

Disease Models & Mechanisms • Supplementary information

**Hybridization chain reaction (HCR) probes**	
sox10 HCR probes	This paper; Molecular Technologies
foxc1b HCR probes	This paper; Molecular Technologies
nf2a HCR probes	This paper; Molecular Technologies
nf2b HCR probes	This paper; Molecular Technologies
mitfa HCR probes	This paper; Molecular Technologies
igfbp2a HCR probes	This paper; Molecular Technologies
**Antibodies**	
NF2/Merlin antibody	GENETEX - Cat No GTX48502
Histone H3 antibody	Abcam - ab1791
Sox10 antibody	GENETEX - Cat No GTX128374
phospho-histone3 (pH3) antibody	Abcam - Cat No ab14955
**Oligonucleotides**	
**PCR primers**	
*nf2a*_gRNA1 (w/o PAM) - GGATCATGATGTGCCCAAAG	This paper; IDT
*nf2a*_gRNA3 (w/o PAM) - GTACGACGTCAAAGACACTG	This paper; IDT

*nf2b*_gRNA1 (w/o PAM) - GGAGGAGAAGATAACCGCT	This paper; IDT
*nf2b*_gRNA2 (w/o PAM) - GGCCAATGGTCCGGCATACC	This paper; IDT
nf2a gR3_exonprimer RP - TTCTTGTCCATTTTCAGCCAGG	This paper; IDT
nf2b gR1_exonprimer FP - TGATGCAGTATCAGATGACACCAG	This paper; IDT
nf2b gR1_exonprimer RP - TTTCTGTGTTCAGCATACCAAGC	This paper; IDT
nf2b gR2_exonprimer FP - TGGAAAGGGAAAGATTTATTTGACC	This paper; IDT
nf2b gR2_exonprimer RP - CAGGCTTCAGCCAGGCAT	This paper; IDT
beta-actin FP – CGAGCTGTCTTCCCATCCA	Tang R et al, Acta Biochimica et Biophysica Sinica,2007; IDT
beta-actin RP - TCACCAACGTAGCTGTCTTTCTG	Tang R et al, Acta Biochimica et Biophysica Sinica,2007; IDT
**T7 endonuclease assay PCR primers**	
nf2b_gR1_FP -	This paper; IDT

Disease Models & Mechanisms • Supplementary information

CGGGGACTACGATCCAAACTT	
nf2b_gR1_RP - ACATGTCTAGATCCTGGGCTAT	This paper; IDT
nf2b_gR2&3_FP - GGGTCTGAAAAAGAAGCAACCAAA	This paper; IDT
nf2b_gR2&3_RP - ATCAGGCTTCAGCCAGGCATA	This paper; IDT
nf2a_gR1_FP - GCAGAGTGCAACACTTATAATGACT	This paper; IDT
nf2a_gR1_RP - GAAACCGGCCCCATGAGTAA	This paper; IDT
nf2a_gR2&3_FP - GTGGCGAGGAAAGGATCTGT	This paper; IDT
nf2a_gR2&3_RP - TTCTTGTCCATTTTCAGCCAGG	This paper; IDT

Disease Models & Mechanisms • Supplementary information
